# Evaluation of the effectiveness and safety of cupping therapy in the treatment of asthma: A protocol for systematic review and meta-analysis

**DOI:** 10.1097/MD.0000000000027518

**Published:** 2021-10-15

**Authors:** Lei Guo, Lie Wang, Zhongtian Wang, Lina Wei, Lizhong Ding, Yibu Kong, Zhimei Liu, Ye Tian, Fushuang Yang, Liping Sun

**Affiliations:** a Changchun University of Chinese Medicine, 1035 Boshuo Road, Changchun City, Jilin Province, China; b Affiliated Hospital of Changchun University of Chinese Medicine, 1478 Gongnong Road, Changchun City, Jilin Province, China.

**Keywords:** asthma, cupping, meta-analysis, protocol, systematic review

## Abstract

**Background:**

Asthma is one of the most common chronic airway diseases and is characterized by wheezing, dyspnea, chest tightness, and coughing. These symptoms reduce the patient's quality of life and limit physical activity in daily life. However, there is no systematic review of the efficacy of cupping therapy in the treatment of asthma. To evaluate the efficacy and safety of cupping in the treatment of asthma, we conducted a systematic review and meta-analysis of published randomized clinical trials of cupping in the treatment of asthma.

**Methods:**

We will search the following Chinese and English databases: China National Knowledge Infrastructure, China Science and Periodical Database, Wanfang Database, China Biomedical Literature Database, PubMed, Embase, Cochrane Library. All of the above electronic databases will be searched from inception to August 22, 2021. In addition, we will manually search for conference papers, ongoing experiments, and internal reports to supplement the studies retrieved via electronic search. We will use the Review Manager 5.4 provided by Cochrane Collaboration Network for statistical analysis.

**Results:**

The study will prove the effectiveness and safety of cupping in the treatment of asthma.

**Conclusion:**

We plan to submit this systematic review to a peer-reviewed journal.

**INPLASY registration number:**

INPLASY202180104.

## 1 Introduction

Asthma is one of the most common chronic airway diseases and is characterized by wheezing, dyspnea, chest tightness, and coughing.^[1]^ These symptoms reduce the patient's quality of life and limit physical activity in daily life.^[2]^ At present, approximately 334 million people worldwide suffer from asthma, and its incidence is increasing rapidly at a rate of 20% to 25% every 10 years. It is estimated that the number of asthma patients will increase to 400 million in 2025, which will lead to a huge health and economic burden in the country.^[3]^ Corticosteroids are conventional drugs for the treatment of bronchial asthma. However, despite the use of medium to high doses of inhaled corticosteroids and long-acting β2 receptor agonists, many patients with severe asthma continue to experience asthma symptoms and worsening, leading to the emergence of refractory asthma.^[4]^ In addition, the long-term use of steroid drugs will bring about a series of adverse reactions, such as local Candida infection, cataracts, glaucoma, osteoporosis, tachycardia, and hyperglycemia.^[5]^ Existing studies have shown that cupping therapy can quickly improve the symptoms of asthma patients who are not sensitive to corticosteroids and bronchodilators and can be used as an alternative therapy.^[6]^ However, there is no systematic review of the efficacy of cupping therapy in the treatment of asthma. To evaluate the efficacy and safety of cupping in the treatment of asthma, we conducted a systematic review and meta-analysis of published randomized clinical trials (RCTs) of cupping in the treatment of asthma. We aim to provide a scientific reference plan for the alternative treatment of asthma.

## 2 Methods and analysis

### 2.1 Information sources and search strategies

The protocol was prepared based on the preferred reporting project of the systematic review and meta-analysis protocol statement guidelines. Our research does not require ethical approval, as all analyses will be based on aggregated data from previously published studies. We will search the following Chinese and English databases: China National Knowledge Infrastructure, China Science and Periodical Database, Wanfang Database, China Biomedical Literature Database, PubMed, Embase, Cochrane Library. We will use “cupping” and “asthma” as keywords to retrieve RCTs examining the treatment of asthma. All of the above electronic databases will be searched from inception to August 22, 2021. In addition, we will manually search for conference papers, ongoing experiments, and internal reports to supplement the studies retrieved via electronic search. The prospective registration has been approved by the International Platform of Registered Systematic Review and Meta-analysis Protocols (https://inplasy.com/inplasy-2021-8-0104/) under registration number inplasy202180104.

### 2.2 Inclusion criteria

The inclusion criteria will be as follows: meeting the clinical diagnostic criteria for asthma, regardless of race, age, and sex; since the establishment of the database, all Chinese and English versions of cupping therapy have been randomized controlled trials in the treatment of asthma; cupping therapy in the treatment group alone or in combination with other therapies, and the control group does not include cupping therapy; research articles that evaluate the maximum peak expiratory flow rate, forced vital capacity, forced expiratory volume in the first second, asthma control questionnaire score, and asthma quality of life questionnaire score.

### 2.3 Exclusion criteria

The exclusion criteria will be as follows: studies that do not meet the above inclusion criteria; incomplete or incorrectly researched data; patients with asthma who have other diseases or serious complications; case reports, comments or letters, biochemical tests, protocols, meeting abstracts, and reviews.

### 2.4 Outcome measures

The main outcome indicators will be peak expiratory flow, forced vital capacity, and forced expiratory volume in the first second, and the secondary outcome indicators will include asthma control questionnaire score, asthma quality of life questionnaire score, and adverse events. Adverse events are defined as negative or unexpected clinical manifestations after treatment, but they are not necessarily causally related to treatment.

### 2.5 Literature screening and data extraction

#### 2.5.1 Literature screening

The literature will be independently screened by 2 researchers (XW and XD) according to the inclusion and exclusion criteria. Then, the data extracted by each researcher will be given to the other party for inspection, and any disagreements will be resolved via discussion. The researchers will first read the titles and abstracts, conduct a preliminary screening, and exclude articles that are not related to the research topic. Then, they will read the full text further to determine whether a study can be included.

Literature screening process: Endnote software will be used to eliminate duplicate literature; the title and abstract of the articles will be read, and irrelevant literature will be excluded; the full text of the remaining articles will be read to determine whether these studies will be included in the study.

#### 2.5.2 Data extraction

The following information will be extracted: research characteristics, including title, first author, research year, and country; basic characteristics of the research object, including number of cases, intervention measures, treatment types, intervention time, outcome indicators, etc; key elements of bias risk assessment; and research results.

### 2.6 Assess the risk of bias in the included studies

Two researchers (YH and JW) will use the RCT bias risk assessment tool recommended by the Cochrane Collaboration Network bias risk assessment tool to assess the risk of bias among the included studies. Disagreements will be resolved by consulting a third researcher.

### 2.7 Assessment of heterogeneity and reporting bias

We will use the standard *I*^2^ test to evaluate statistical heterogeneity; *I*^2^ < 50% indicates that there is no significant heterogeneity, and *I*^2^ ≥ 50% indicates significant heterogeneity. We will use a funnel chart to assess publication bias.

### 2.8 Statistical analysis of data

We will use RevMan 5.4 software (Cochrane Collaboration) for the meta-analysis. We will use relative risk as an effective indicator of counting data and use the mean differences as an effective indicator of measurement data. The confidence interval for each effect index will be set to 95%. Additionally, *I*^2^ will be used to quantitatively assess heterogeneity. If there is no statistical heterogeneity between the studies, a fixed effects model will be used for the meta-analysis. If there is heterogeneity, a random effects model will be used. *P* < .05 indicates statistical significance.

### 2.9 Analysis of subgroups or subsets

When there is potential heterogeneity in this study, if all the information included in the study was available, we could perform subgroup analysis based on the sex, age, and treatment time of the included subjects.

### 2.10 Sensitivity analysis

A sensitivity analysis will be performed to assess the robustness of the included results. If the results are unstable, studies with a high risk of bias will be excluded.

### 2.11 Ethics and publishing

These data are obtained from the database and do not require ethical approval. We will submit the final research results to a peer-reviewed journal for publication.

## 3 Discussion

Cupping is an external treatment method of Chinese medicine, with thousands of years of application history. As early as 1931, Osler pointed out that cupping can treat bronchial pneumonia and acute myelitis.^[7]^ In recent years, cupping therapy has become very popular among Western athletes, such as Michael Phelps, Alex Naddour, and Pavel Sankovich, because cupping can relieve muscle fatigue and reduce related inflammation indicators after exercise.^[8,9]^ In the clinic, cupping therapy has been proven to have potential benefits for low back pain, ankylosing spondylitis, knee osteoarthritis, neck pain, herpes zoster, migraine, plaque psoriasis, and chronic urticaria, as well as asthma.^[10,11]^ Asthma has been the focus of global public health and has received increasing attention. However, there are still relatively few studies on cupping therapy in the treatment of asthma, and there is not enough evidence to explain the clinical efficacy of cupping therapy in the treatment of asthma. Therefore, this systematic review and meta-analysis will evaluate the effectiveness and safety of cupping therapy for asthma. It is hoped that this study will provide clinical evidence-based evidence for the treatment of asthma by cupping therapy.

## Author contributions

**Conceptualization:** Lei Guo, Zhongtian Wang.

**Data curation:** Lei Guo, Ye Tian, Fushuang Yang.

**Formal analysis:** Lei Guo, Ye Tian, Fushuang Yang.

**Funding acquisition:** Lei Wang, Liping Sun.

**Investigation:** Lizhong Ding, Zhimei Liu.

**Methodology:** Lina Wei, Yibu Kong.

**Project administration:** Lizhong Ding, Zhimei Liu.

**Resources:** Lina Wei, Yibu Kong.

**Software:** Lizhong Ding, Yibu Kong, Zhimei Liu.

**Supervision:** Lei Wang, Liping Sun.

**Validation:** Lei Guo, Zhongtian Wang.

**Visualization:** Zhongtian Wang, Lina Wei, Zhimei Liu.

**Writing – original draft:** Lei Guo.

**Writing – review & editing:** Lei Guo, Lei Wang, Liping Sun.
